# Human Innate Mycobacterium tuberculosis–Reactive αβTCR^+^ Thymocytes

**DOI:** 10.1371/journal.ppat.0040039

**Published:** 2008-02-15

**Authors:** Marielle C Gold, Heather D Ehlinger, Matthew S Cook, Susan K Smyk-Pearson, Paul T Wille, Ross M Ungerleider, Deborah A Lewinsohn, David M Lewinsohn

**Affiliations:** 1 Department of Pulmonary and Critical Care Medicine, Portland VA Medical Center, Oregon Health and Science University, Portland, Oregon, United States of America; 2 Department of Pediatrics, Oregon Health and Science University, Portland, Oregon, United States of America; 3 Department of Molecular Microbiology and Immunology, Oregon Health and Science University, Portland, Oregon, United States of America; 4 Doernbecher Cardiothoracic Surgery, Doernbecher Children's Hospital, Portland, Oregon, United States of America; Johns Hopkins School of Medicine, United States of America

## Abstract

The control of Mycobacterium tuberculosis (Mtb) infection is heavily dependent on the adaptive Th1 cellular immune response. Paradoxically, optimal priming of the Th1 response requires activation of priming dendritic cells with Th1 cytokine IFN-γ. At present, the innate cellular mechanisms required for the generation of an optimal Th1 T cell response remain poorly characterized. We hypothesized that innate Mtb-reactive T cells provide an early source of IFN-γ to fully activate Mtb-exposed dendritic cells. Here, we report the identification of a novel population of Mtb-reactive CD4^−^ αβTCR^+^ innate thymocytes. These cells are present at high frequencies, respond to Mtb-infected cells by producing IFN-γ directly ex vivo, and display characteristics of effector memory T cells. This novel innate population of Mtb-reactive T cells will drive further investigation into the role of these cells in the containment of Mtb following infectious exposure. Furthermore, this is the first demonstration of a human innate pathogen-specific αβTCR^+^ T cell and is likely to inspire further investigation into innate T cells recognizing other important human pathogens.

## Author Summary

Mycobacterium tuberculosis (Mtb) infects about one-third of the world's population. Most people who are exposed remain healthy, but control of this intracellular bacterium requires a robust cellular immune response. Production of the pro-inflammatory cytokine IFN-γ from cells in the adaptive immune response is critically important in the immune control of Mtb*.* However, this cytokine is also essential in initiating an optimal adaptive immune response. We hypothesized that innate cells could provide an early source of IFN-γ to aid in generation of an optimal adaptive immune response. We looked for IFN-γ producing cells in human neonates that were unlikely to have been previously exposed to either Mtb or other environmental mycobacteria. Here, we report the identification of a novel T cell population from the thymus that produces IFN-γ in response to Mtb-infected cells. Mtb-reactive thymocytes are present at high frequencies, are present in nearly all newborns tested, and display characteristics of T cells normally associated with a memory response. This novel innate population of Mtb-reactive cells will drive further investigation into the role of these cells in the containment of Mtb following infectious exposure and is likely to inspire further investigation into innate T cells recognizing other important human pathogens.

## Introduction

Approximately one-third of the world's population is infected with Mycobacterium tuberculosis (Mtb). Tuberculosis remains a leading cause of mortality worldwide and is responsible for 2–3 million deaths per year [1]. Household contact studies show that over 50% of exposed individuals never develop a positive tuberculin skin test (TST), and are considered uninfected [2]. However, in those who have been detectably infected with Mtb, as assessed by a positive TST, the lifetime risk of tuberculosis is estimated at 5% to 10% [3]. In the remaining 90% of TST-positive individuals, protection is associated with the development of an effective adaptive immune response. Specifically, Th1-like cytokines, including IFN-γ, produced by CD4^+^ Th1 cells and CD8^+^ T cells, and TNF-α , produced by T cells and macrophages, are essential in the control of tuberculosis [4].

The priming of Mtb-specific naïve T cells requires that dendritic cells (DC), either infected with Mtb or having engulfed Mtb-derived antigens, migrate to the lymph nodes [5,6]. There, to optimally prime pathogen-specific Th1 responses, DC require stimulation through Toll-like receptors (TLRs) [7] by the pathogen as well as host-derived factors such as type I and type II IFNs, cytokines, and chemokines [8].

Mtb-dependent TLR2 ligation can promote the maturation of DC via upregulation of costimulatory molecules and the production of IL-12 [9,10]. However, in comparison to potent LPS activation associated with TLR-4 ligation, Mtb induces relatively low levels of IL-12 production [11,12]. Nonetheless, IL-12 production by DC is essential to prime optimal Th1 responses [9,13–15]. IFN-γ, the prototypical Th1 cytokine, can directly augment the IL-12 production. In addition to NK cells, MHC Ib-restricted T cells are able to provide an early source of IFN-γ for enhanced IL-12 production by DC [9,14,15]. One example of these innate T cells comes from the study of the non-classical MHC-Ib murine molecule H2-M3. Pamer and colleagues defined H2-M3 restricted, *Listeria*-specific, IFN-γ-producing T cells, whose response preceded that of the adaptive T cell response and was therefore consistent with an innate T cell population [16]. Subsequently, Urdahl et al. demonstrated the presence of MHC Ib-restricted cells in antigen-naïve mice and that found that they originated from the thymus and displayed properties associated with effector T cells [17].

In this study we sought to test the hypothesis that humans possess an innate population of Mtb-reactive T cells. From this hypothesis, we made the following predictions: 1) the innate T cell population would, unlike a naïve T cell precursor population, be present at high frequency in the circulating pool of lymphocytes; 2) the innate T cell population could respond directly ex-vivo to Mtb-infected cells in the absence of clonal expansion; 3) these cells would be prevalent throughout the human population irrespective of prior exposure to mycobacteria; and 4) these cells would be thymically derived.

Here we show that Mtb-reactive thymocytes are present at frequencies ranging from 0.01% to 0.2% of CD4^−^ thymocytes in humans. The majority of Mtb-reactive thymocytes express the αβTCR and an activated phenotype with cytolytic potential. Mtb-reactive thymocytes require cell contact with DC infected with live Mtb and respond through a mechanism most consistent with MHC class Ib–restricted T cells. Consistent with the hypothesis that humans contain innate T cells that are capable of responding to Mtb-infected DC, we have identified a population of Mtb-reactive cells in cord blood.

## Results

### Human CD4^−^ Mtb-Reactive Thymocytes Are Present at High Frequency in the Thymus

**Figure 1:**
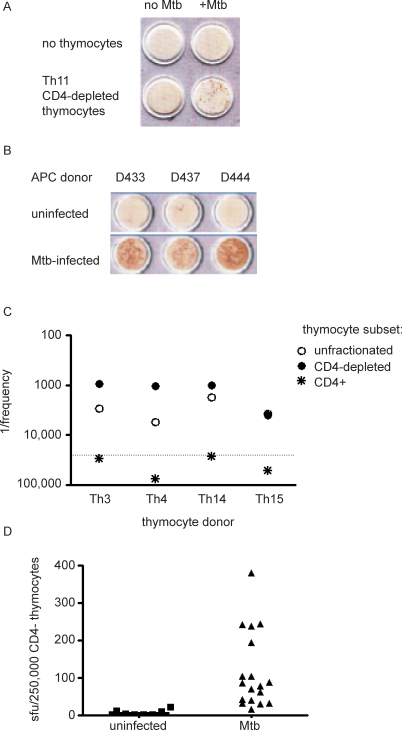
Mtb-Reactive CD4^−^ Thymocytes Are Present in the Human Thymus (A) CD4-depleted thymocytes from thymocyte donor 11 (750,000/well) were incubated overnight with autologous DC (20,000/well) that were either infected with Mtb (moi of 50) or left uninfected. (B) Thymocytes (250,000 cells/well) from one thymocyte donor were incubated with MHC mismatched DC (50,000 cells/well) from 3 different donors. (C) Thymocytes from 4 individual donors were left unfractionated, or fractionated based on the positive or negative expression of CD4 using CD4 magnetic bead separation and incubated overnight with Mtb-infected DC (50,000/well). The dotted line represents the limit of detection of the assay. The lack of Mtb-reactivity from the CD4^+^ thymocytes fraction has been reproducible and repeated with over 10 donors. (D) CD4-depleted thymocytes (500,000/well) were titrated in a series of 2-fold dilutions and incubated with Mtb-infected DC (50,000/well). Frequencies were determined by linear regression analysis as described in Materials and Methods. In all experiments, IFN-γ production was assessed by ELISPOT. Mtb-reactive thymocytes (*n* = 18) ranged from 16.5 to 381 spot-forming units (sfu)/250,000 CD4^−^ thymocytes with a mean of 115.9 ± SD 101.2.

To assess whether or not humans contain a population of innate T cells that can respond to Mtb-infected cells we used thymocytes from infants undergoing cardiac surgery where thymectomy is standard procedure. Based on observations from Pamer and Urdahl, we speculated that such a population would be found in the CD4-negative population of thymocytes. Therefore, we used magnetic beads to deplete thymocytes of CD4^+^ cells. The CD4-depleted population was then incubated with autologous monocyte-derived Mtb-infected DC. We enumerated IFN-γ production from thymocytes by IFN-γ ELISPOT. Thymocytes from one donor produced IFN-γ in response to autologous DC infected with Mtb but not to uninfected DC while no IFN-γ was detected from Mtb-infected DC alone (Figure 1A).

We reasoned that these cells were not likely to be classically HLA-Ia restricted. HLA class Ib molecules, unlike the highly polymorphic HLA class Ia molecules, have limited polymorphism, such that most individuals will express a similar set of these molecules. As a result, we predicted that allogeneic DC would serve as antigen-presenting cells (APC) for these T cells. Allogeneic DC were tested for their ability to elicit IFN-γ production by thymocytes. CD4-depleted thymocytes incubated with allogeneic DC produced IFN-γ in response to Mtb-infected DC but not to uninfected DC from three separate allogeneic donors (Figure 1B). Further evaluation of over 40 thymocyte donors using allogeneic DC revealed detectable IFN-γ responses to the Mtb-infected allogeneic DC but not to the uninfected DC (data not shown). The finding that thymocytes are consistently unresponsive to uninfected allogeneic DC is in sharp contrast to the predictable response by peripheral T cells that exhibit strong alloreactivity in response to MHC mismatched DC. Fortuitously, the absence of alloreactivity provided us with the opportunity to employ allogeneic DC given the limited quantities of PBMC available from the thymocyte donors. We confirmed that in response to allogeneic Mtb-infected DC the Mtb-reactive thymocytes are present in the CD4-negative but not in the CD4^+^ fraction of thymocytes (Figure 1C). CD8^+^ T cells from peripheral blood are distinct from Mtb-specific CD4^+^ T cells in that CD8^+^ T cells preferentially recognize APC in direct proportion to the degree of intracellular infection [18]. Similarly, Mtb-reactive thymocytes also preferentially recognize DC infected with Mtb at multiplicities of infection (MOI) of 30 or higher (Figure S1). Nevertheless, greater than 90% of DC infected at an MOI of 30 for 18 h of infection, remain viable, as assessed by trypan blue exclusion (not shown) and a minority (less than 20%) are apoptotic as assessed by Annexin V staining (Figure S2). We next sought to establish the frequency and prevalence of Mtb-reactive thymocytes. Ex vivo frequencies of Mtb-reactive thymocytes (*n* = 18) ranged from 16.5 to 381 sfu/250,000 CD4^−^ thymocytes (mean = 115.9 ± SD 101.2) (Figure 1D). Furthermore, we have detected Mtb-reactive thymocytes from 58 of 60 donors tested (not shown) demonstrating that Mtb-reactive thymocytes are prevalent in humans.

### While γδTCR^+^ Thymocytes Can Be Detected, the Majority of Mtb-reactive Thymocytes Express the αβTCR

**Figure 2:**
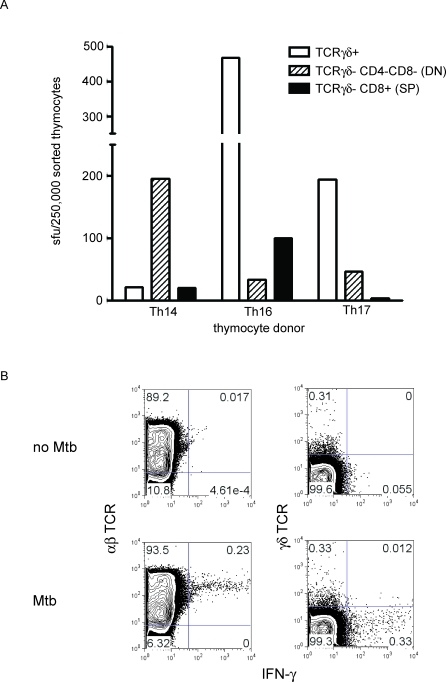
The Majority of Mtb-Reactive Thymocytes Are αβTCR^+^ T Cells, While a Minority Express the γδTCR (A) Thymocytes from 3 random donors were stained with the following fluorochrome-conjugated antibodies: CD4-PE, CD8-APC, γδTCR-FITC. CD4-negative subsets (γδTCR^+^; γδTCR^−^ CD8^+^; γδTCR^−^ CD8^−^) were collected by FACS. Cell subsets were incubated with Mtb-infected DC or uninfected DC in an IFN-γ ELISPOT assay and the response to Mtb-infected DC is shown. No responses to uninfected DC were detected. (B) Unfractionated thymocytes (500,000 cells/well) were incubated with either uninfected or Mtb-infected DC (50,000 cells/well) and IFN-γ production was detected using the ICS assay. Cells were fixed and permeabilized and stained with fluorochrome-conjugated antibodies to label the following: γδTCR, αβTCR, IFN-γ, and CD3. All cells depicted in (B) are CD3^+^. Similar results were obtained from 3 separate donors.

In human peripheral blood, 50% to 90% of all γδ T cells are Mtb-reactive. These T cells primarily express the Vγ9Vδ2 TCR and can respond to the mycobacterial non-peptide antigen isopentenyl pyrophosphate (IPP) [19]. Furthermore, Kabelitz et al. previously described the presence of Vγ9-Mtb-reactive thymocytes in humans [20]. Thus, it was conceivable that γδTCR-expressing cells constituted the majority of Mtb-reactive thymocyte responses. Therefore, thymocytes were sorted by FACS by selecting CD4-negative cells that expressed CD8 and/or the γδTCR. The subsets were tested on Mtb-infected and uninfected DC. None of the sorted cells produced IFN-γ in response to uninfected DC (not shown). Figure 2A shows that both γδTCR^−^ and γδTCR^+^ thymocytes respond to Mtb-infected DC and that as expected, a high frequency of thymocytes that express the γδTCR can respond to Mtb-infected DC. However, both CD8 single positive (SP) and CD4^−^CD8^−^ double negative (DN) thymocytes in the γδTCR-depleted subset also contained Mtb-reactive thymocytes. Therefore, we used the intracellular cytokine staining (ICS) assay with unfractionated thymocytes to determine if the γδTCR-negative cells expressed the αβTCR. Figure 2B shows that CD3^+^ IFN-γ^+^ Mtb-reactive thymocytes detected by ICS express the αβTCR and not the γδTCR. The fact that we did not detect γδTCR^+^ IFN-γ^+^ Mtb–reactive cells by ICS is consistent with the observation that less than 1% of all thymocytes express the γδTCR [21]. Furthermore, these results indicate that although Mtb-reactive T cells expressing either the γδ or αβ TCR are both present in the thymus, the vast majority of Mtb-reactive thymocytes are αβTCR^+^ CD4^−^ T cells that are either CD8^+^ SP or DN.

### Mtb-Reactive Thymocytes Express an Activated Effector Phenotype

**Figure 3:**
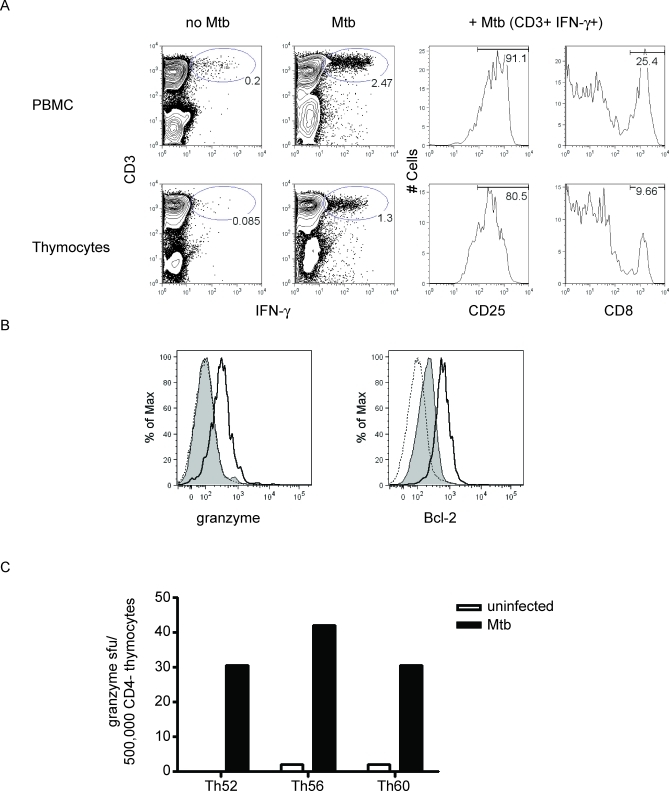
Mtb-Reactive Thymocytes Display Molecules Associated with an Activated Effector Phenotype (A) Unfractionated thymocytes (500,000 cells/well) and positive control PBMC (500,000 cells/well) were incubated with allogeneic DC (50,000 cells/well) infected with Mtb or left uninfected and IFN-γ assessed using the ICS assay. Non-specific binding by the mouse IgG1 PE isotype was not observed. The numbers in the graphs represent the percentage of IFN-γ-positive cells of unfractionated cells. Histograms represent expression of CD25, and CD8 on gated CD3^+^IFN-γ^+^ cells after stimulation with Mtb. (B) Histograms depicting granzyme and Bcl-2 expression from CD3^+^IFN-γ^+^-gated cells after stimulation with Mtb. Dotted line: isotype control antibody; Shaded histogram: granzyme or Bcl-2 expression on total CD3^+^ thymocytes; Bold line: granzyme or Bcl-2 expression on IFN-γ^+^ CD3^+^ cells (Mtb-reactive). Data are representative of a minimum of 4 thymocyte donors. (C) CD4-depleted thymocytes (500,000 cells/well) from 3 individuals were tested for their ability to secrete granzyme in an ELISPOT assay in response to DC (50,000 cells/well) that were uninfected or infected overnight with Mtb (moi 30).

Mtb-reactive thymocytes, by virtue of producing IFN-γ directly ex vivo inherently display a Th1-type effector phenotype. To further characterize the phenotype of Mtb-reactive thymocytes we used the ICS assay to assess the expression of molecules associated with effector memory T cells. As a positive control we used PBMC from an adult donor with known T cell reactivity to mycobacterial antigens. PBMC or Mtb-reactive thymocytes were incubated with allogeneic DC infected with Mtb or left uninfected. As expected, uninfected allogeneic DC induced a detectable frequency of PBMC to produce IFN-γ while a much lower frequency was detected from thymocytes. Mtb-reactive cells were defined by the specific production of IFN-γ in response to Mtb-infected DC. All Mtb-reactive thymocytes expressed CD3. This confirms the commitment of these cells to the T cell lineage (Figure 3) [22]. Furthermore, no Mtb-reactive cells expressed CD161 (data not shown), a marker expressed on both immature and mature NK cell cells in the thymus [23] further confirming that these cells are not NK cells. Consistent with results shown in Figure 2A, a proportion of the Mtb-reactive thymocytes expressed CD8. The large majority of Mtb-reactive thymocytes expressed CD25 (Figure 3A, right), and granzyme (Figure 3B), as well as TNF-α (not shown) consistent with an effector memory phenotype with cytolytic potential [24]. Furthermore, a proportion of CD4^−^ thymocytes secreted granzyme in response to Mtb-infected DC (Figure 3C). Finally, Mtb-reactive thymocytes expressed higher levels of Bcl-2 (Figure 3B) suggesting these cells are likely to survive and egress to the periphery [25].

### Mtb-Reactive Thymocytes Are Stimulated by DC Infected with Live Mtb But Not DC Incubated with TLR Agonists

**Figure 4:**
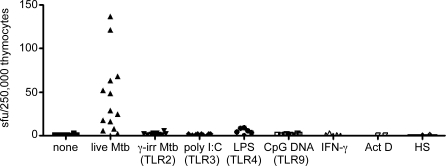
Mtb-Reactive Thymocytes Are Stimulated by DCs Infected with Live Mtb but Not DCs Incubated with TLR Agonists DC were incubated overnight with TLR agonists specific for TLR2 (γ-irradiated Mtb, moi equivalent of 500), TLR3 (poly I:C; 50 μg/ml), TLR4 (LPS; 100 ng/ml) TLR9 (CpG DNA; 6 μg/ml) or treated with IFN-γ (10 ng/ml), or Actinomycin D (10 μM) or heat shock-treated (42°C for 90 min) and were tested for their ability to stimulate DC to elicit IFN-γ production by thymocytes in comparison to live Mtb-infected DC (moi 30). Unfractionated thymocytes (250,000 cells/well) were incubated with the DC (50,000 cells/well) and tested for their ability to produce IFN-γ in an ELISPOT assay.

Next, we tested the hypothesis that TLR-activation of DC, or other stimuli, may be sufficient to stimulate thymocytes. Mtb principally acts through TLR2 [26–29], TLR9 [29], and to a lesser extent TLR4 [30]. Therefore, we tested IFN-γ production by thymocytes incubated with DC pre-treated with agonists to TLR2 (γ-irradiated Mtb), TLR4 (LPS), TLR9 (CpG DNA), and TLR3 (poly I:C). None of these TLR stimuli induced a comparable response to that elicited by live Mtb infection of DC (Figure 4). We confirmed that treatment of DC with the γ-irradiated Mtb and LPS indeed functioned as TLR agonists by inducing IL-10 production from the DC (Figure S3). Furthermore, an additional TLR2 agonist, lipoteichoic acid, did not induce IFN-γ production by thymocytes (not shown). To test whether or not infection of DC may be triggering the expression of molecules associated with cellular stress, we incubated DC with IFN-γ, actinomycin D, or heat shock-treated the DC. CD4^−^ thymocytes did not respond to the stress-induced DC. We confirmed that actinomycin D indeed induced apoptosis in over 55% of the DC as assessed by Annexin-V (Figure S2).

### Activation of Mtb-Reactive Thymocytes Requires Cell Contact with Mtb-Infected DC

**Figure 5:**
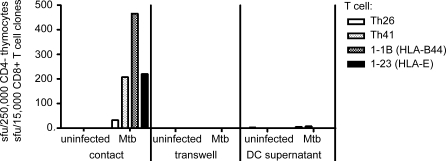
Mtb-Reactive Thymocytes Require Cell Contact with Mtb-Infected DC to Produce IFN-γ CD4-depleted thymocytes (250,000 cells/well) were tested for their ability to produce IFN-γ in an ELISPOT assay in response to uninfected or Mtb-infected DC (50,000 cells/well) that were either placed directly in contact with the T cells (contact) on the ELISPOT membrane or placed in the upper wells of a 96-well Transwell plate (0.4-μm pore size) above the ELISPOT plate (transwell). Supernatants (100 μl) from uninfected or Mtb-infected DC were added directly to the T cells (supernatant). CD8^+^ T cell clones (15,000 cells/well) were used as positive controls for the response to Mtb-infected DC. Comparable results were obtained in 3 separate experiments.

To help elucidate the mechanism by which Mtb-reactive thymocytes respond to Mtb-infected DC, we asked if thymocytes require direct contact with Mtb-infected targets. The presence of a transwell between Mtb-infected DC and thymocytes prevented IFN-γ release (Figure 5), demonstrating that direct contact of thymocytes with Mtb-infected DC is required. In addition, cell supernatants from Mtb-infected DC did not induce IFN-γ production by thymocytes suggesting that cytokines produced from Mtb-infected DC are not sufficient for this response.

### Activation of Mtb-Reactive Thymocytes Is Proteasome Dependent But Is Not Blocked by the Pan–HLA-I Antibody W6/32

**Figure 6:**
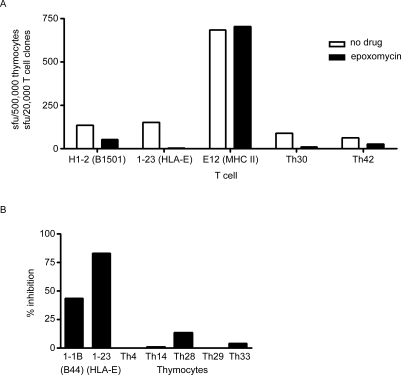
Activation of Mtb-Reactive Thymocytes Is Proteasome Dependent but Is Not Blocked by the Pan–HLA-I Antibody W6/32 (A) DC were pretreated for 1 h with epoxomycin (1 μM). The DC were then infected with Mtb (moi 25). After 24 h, the drug was washed away and DC were fixed with cold paraformaldehyde (0.1%) for 5 min, washed twice in PBS, incubated for 2 h in media, and then washed three times. The DC (50,000 cells/well) were added to T cell clones (5000 cells/well) or CD4-depleted thymocytes (250,000 cell/well). Comparable results were obtained in 3 separate experiments. (B) Mtb-infected DC were incubated with the W6/32 antibody (2 μg/ml) or a mouse IgG2a isotype control (2 μg/ml) for 15 min before the addition of CD8^+^ T cell clones (10,000/well) or CD4-depleted thymocytes (250,000/well). IFN-γ production was evaluated by ELISPOT. The percent inhibition was calculated from the response to Mtb-infected DC in the presence of the W6/32 blocking antibody divided by the response to Mtb-infected DC in the presence of the isotype control.

To further evaluate the possibility that Mtb-reactive thymocytes respond to an antigen processed and presented through the HLA-I pathway versus to a cell surface ligand upregulated by live infection with Mtb, we used blocking antibodies to molecules induced by infection with Mtb. We were unable to block responses by Mtb-reactive thymocytes (*n* = 5) using antibodies against MICA or ULBP1 [31] or the receptor NKG2D [32] (not shown). We then blocked proteasomal function to prevent production of HLA class I–restricted Mtb epitopes. Addition of the proteasomal blocker, epoxomycin, blocked 88% of the response by Mtb-reactive thymocytes from donor Th30 and 57% of the response from donor Th42 (Figure 6A). As expected, the CD8^+^ T cell clones H1–2 (restricted by HLA-B1501) and 1–23 (restricted by HLA-E) were also blocked by epoxomycin (62% and 97% respectively) while the CD4^+^ T cell clone E12 (restricted by HLA-II) was not blocked. Nevertheless, we have been unsuccessful in blocking the response by Mtb-reactive thymocytes using a variety of blocking antibodies to HLA-Ia and HLA-Ib molecules. Addition of the pan–HLA-I blocking antibody W6/32 did not inhibit responses by the thymocytes (Figure 6B). In contrast, the HLA-B44– and HLA-E–restricted CD8 T cell clones were effectively blocked. Addition of blocking antibodies to the nonclassical CD1a, b, c, and d molecules also did not prevent responses by Mtb-reactive thymocytes (data not shown). Thus, our data suggest that antigen processing is likely required for activation of at least a subset of thymocytes. As paraformaldehyde-fixed cells were used as the APCs in these experiments, an additional conclusion from these data, in combination with results from Figure 5, is that a cell surface ligand and not a soluble factor is required to stimulate Mtb-reactive thymocytes. Thus, while ligand interaction is required, the cells are not restricted by HLA-Ia, HLA-E, or CD1 molecules.

### Mtb-Reactive Cells Are Present in Cord Blood

**Figure 7:**
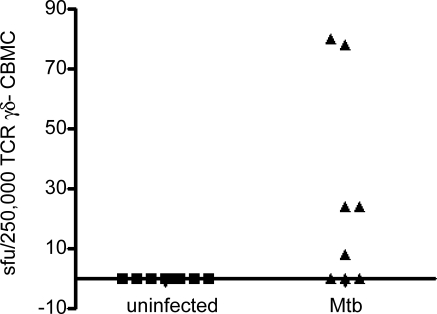
Mtb-Reactive Cells Are Present in Cord Blood Cord blood mononuclear cells (250,000 cells/well) were depleted of γδTCR^+^ cells using magnetic bead separation. γδTCR–depleted CBMC were incubated with autologous DC (50,000 cells/well) that were either Mtb-infected (moi = 30) of left uninfected. IFN-γ production was tested in an ELISPOT assay (*n* = 8; range, 0–80 sfu/250,000 γδTCR-depleted CBMC; mean = 26.75 ± SD 33.75).

Physiologically, the relevance of Mtb-reactive thymocytes rests in their ability to egress the thymus, and serve as innate effectors. To address this, we used cord blood mononuclear cells (CBMC) isolated from healthy neonates, as a source of T cells that are naïve to exposure to mycobacterial antigens. The majority of CBMC samples, depleted of γδTCR-positive cells using magnetic beads, produced IFN-γ in response to Mtb-infected DC in an ELISPOT assay (*n* = 8; range, 0–80 sfu/250,000 γδTCR-depleted CBMC; mean = 26.75 ± S.D. 33.75) (Figure 7).

## Discussion

In this study, we find that the human thymus contains cells that recognize Mtb-infected cells. As such, we postulate that these cells comprise an innate defense against mycobacterial infection. Several observations support this hypothesis. The frequencies of Mtb-reactive thymocytes are substantial (0.01% to 0.2% of CD4^−^ thymocytes) and appear similar to frequencies of other innate T cells that mediate immediate responses [33]. For example, MHC class Ib T22-restricted γδTCR^+^ T cells are present at frequencies as high as 0.5% of γδTCR^+^ splenocytes in uninfected mice [34]. In this regard, we note that corticosteroids are frequently administered to the thymus donors in the peri-operative period. As a result, we believe that our results likely underestimate the prevalence of these cells in the thymus.

In contrast to the delayed responses inherent in the requisite proliferation, differentiation, and clonal expansion of adaptively acquired immunity, the thymocytes described herein exhibit rapid effector function characterized by the release of IFN-γ, TNF-α, and constituents of the cytolytic granule. Mtb-reactive thymocytes, by virtue of their ability to produce IFN-γ directly ex vivo, display the phenotype of pre-armed effector Th1-like cells [24]. The rapid production of IFN-γ by T cells is normally induced as a consequence of cell division and differentiation, and is associated with effector and memory, but not naïve T cells [35]. The phenotype of Mtb-reactive thymocytes is reminiscent of cells described by Urdahl et al. who showed that MHC class Ib–restricted T cells with an activated effector phenotype could be isolated from the thymus of naïve mice [16,17]. Therefore, Mtb-reactive thymocytes may represent a subset of innate T cells with direct ex vivo effector function in humans.

Mtb-reactive thymocyte responses are present in the absence of prior antigenic exposure. In children, exposure to environmental mycobacteria occurs as children begin to explore their environment [36]. As most of our thymus donors are very young (all are <4 mo old and many are less than 1 wk old) it is unlikely they have been exposed to environmental mycobacteria. Furthermore, exposure to tuberculosis is very unlikely in our patient population. In Oregon, the 2006 case rate of tuberculosis was 2.2/100,000 individuals (Oregon DHS). Moreover, in our limited experience, we have not found evidence for reactivity to Mtb-specific antigens such as CFP-10 and ESAT-6 (not shown), arguing against the possibility of prior exposure to Mtb.

We find it unlikely that Mtb-reactive T cells in the thymus reflect mature peripheral T cells that have recirculated back to the thymus. While mouse studies have demonstrated the capability of peripheral T cells to recirculate to the thymus [37,38] the injection of substantial numbers of T cells was required to detect this phenomenon [37,39]. Moreover, studies by Fink et al. showed that this required exposure to antigen [38]. As discussed above, it is unlikely that the very young donors described in this report have had prior mycobacterial exposure.

The expression of CD3, and the absence of CD161, demonstrate it is unlikely that the Mtb-reactive thymocytes are NK cells [23]. Furthermore, Mtb-reactive thymocytes do not express Vα24 (not shown) and are therefore not the well-defined subset of invariant NKT cells [40]. However, it is possible that these innate cells are non-invariant TCR NKT cells or those restricted by an HLA-Ib molecule. These hypotheses are not mutually exclusive. Innate-like T cells often recognize a signature antigen in pathogen-infected cells. With regard to the possibility that these cells are NKT cells, it is possible that they recognize a danger signal induced by Mtb in the infected cell that would allow for NK-like recognition. In this regard, we note that blocking of the known NK receptor NKG2D and NK and γδ T cell ligands MICA [32], and ULBP1 [31], did not abrogate the recognition by Mtb-infected cells (data not shown). Alternately, antigen presented in the context of an MHC class Ib-molecule may result in T cell activation. Our data support the hypothesis that Mtb-reactive thymocytes are MHC class Ib-restricted. Mtb-reactive thymocytes are activated by allogeneic Mtb-infected DC and activation requires live infection of DC with Mtb as well as proteasomal processing. As a result, we conclude that Mtb-reactive thymocytes represent a subset of T cells that are most likely MHC class Ib-restricted but may utilize a novel mechanism to detect Mtb-infected DC.

This report provides the first demonstration of a human innate pathogen-reactive αβTCR^+^ T cell. In preliminary experiments we have begun to assess the reactivity of thymocytes to other pathogens (not shown). We have detected modest responses to Staphylococcus aureus–*, E.coli*–*, and Mycobacterium smegmatis*–infected DC but did not detect any responses to Listeria monocytogenes– or vaccinia virus–infected DC. Thus, it is possible that innate thymocytes provide early and innate Th1-like immunity at the site of infection with Mtb and perhaps other pathogens. Furthermore, we have identified Mtb-reactive cells in cord blood. This finding is consistent with the potential egress from the thymus of αβTCR^+^ Mtb-reactive cells. Through the production of IFN-γ, Mtb-reactive cells may act on Mtb-infected macrophages early in infection and as such control the spread of Mtb. It is known that over half of exposed individuals never convert their TST [2]. Therefore, perhaps innate responses allow the clearance of the bacterium and obviate the need for adaptive immunity. Furthermore, IFN-γ from Mtb-reactive cells may provide help to DC to augment the production of IL-12 resulting in an enhanced Th1 response. As such, innate Mtb-reactive cells could act as a bridge between the innate and adaptive responses to Mycobacterium tuberculosis. These findings may inspire further investigation into innate T cells recognizing other important human pathogens.

## Materials and Methods

### Human subjects.

All tissue and blood were obtained under protocols approved by the Institutional Review Board at Oregon Health and Science University. Human thymuses were obtained from children undergoing cardiac surgery at Doernbecher Children's Hospital. The majority of children were less than 1 mo of age and all were less than 4 mo old. However, due to the fact that thymuses were obtained as de-identified medical waste under an exempt IRB protocol no additional information is available on the status of the donors. PBMC were obtained by aphaeresis from normal adult donors with informed consent. Umbilical cord blood was obtained from healthy full-term neonates, collected into CPT tubes (BD) and CBMC were obtained after centrifugation.

### Mycobacterium tuberculosis.

The H37Rv strain of Mycobacterium tuberculosis was used for all live Mtb infections and for experiments using γ-irradiated Mtb (Mycobacteria Research Laboratories at Colorado State University).

### Cells.

*Thymocytes:* Thymus tissue was cut into 3-mm^3^ pieces. Each piece was ground in a Medimixer with 1 ml of DMEM plus 10% FBS to form a single cell suspension. The suspension was cryopreserved at 2 × 10^8^ cells/ml in a 90% FBS/10% DMSO freezing solution with a post-thaw viability of approximately 50%. To deplete CD4^+^ thymocytes we positively selected CD4^+^ cells using magnetic bead separation according to the manufacturer's instructions (Miltenyi) and used the remaining untouched cells that contain CD8^+^ (SP) and CD8^−^CD4^−^ (DN) cells. The CD4^+^ selection procedure resulted in a population of cells with a mean purity of 80% CD4-negative cells (range, 60% to 95% CD4-negative cells; not shown).

*Monocyte-derived DC:* Monocyte-derived DCs were prepared according to the method by Romani et al. [41]. Briefly, PBMC or CBMC were resuspended in 2% human serum (HS) medium and allowed to adhere to a T-75 (Costar) flask at 37°C for 1 h. After gentle rocking, nonadherent cells were removed and 10% HS medium containing 10 ng/ml of IL-4 (Immunex) and 30 ng/ml of GM-CSF (Immunex) was added to the adherent cells. After 5 d, cells were harvested with cell-dissociation medium (Sigma-Aldrich) and used as APC in assays.

### Assays.

*IFN-γ ELISPOT assay:* All IFN-γ ELISPOT assays were performed as previously described [42]. Thymocytes were incubated for 24 h with DC that were previously infected overnight with Mtb H37Rv. A range of multiplicity of infection of 25 to 50 was used throughout our studies.

*Estimation of the frequency of Mtb-reactive thymocytes using the IFN-γ ELISPOT:* Thymocytes were added to ELISPOT plates in duplicate over a range of concentrations (5 × 10^5^, 2.5 × 10^5^, 1.25 × 10^5^, 6.25 × 10^4^ cells/well) with DC (50,000 cells/well) infected with Mtb or left uninfected. For determination of effector cell frequencies, the general estimating equation in the GraphPad Prism 3.0 software package was used. If the control frequencies were determined to be significantly different (*p* < 0.05) from the treatment group, control values were subtracted out to determine the frequencies of Mtb-reactive thymocytes.

*Intracellular cytokine staining assay:* Thymocytes (500,000/well) were added to DC (50,000/well) that were either Mtb-infected or uninfected and incubated for 48 h in the presence of anti-CD28 (1 μg/ml) and CD49d (1 μg/ml). GolgiStop (BD Pharmingen) was added for the final 6 h of the assay. Cells were fixed with paraformaldehyde (final 1%), permeabilized with Perm/Wash (BD Pharmingen), and stained with fluorochrome-conjugated antibodies to both IFN-γ and cell surface receptors. Acquisition was performed with an LSRII flow cytometer with FACS Diva software. All analyses were performed using FlowJo software (TreeStar).

*IL-10 ELISPOT assay:* The Human IL-10 ELISpot PLUS kit (ALP) was used to detect IL-10 and performed according to the manufacturer's instructions (Mabtech).

*Granzyme ELISPOT assay:* The BD ELISpot human granzyme B kit was used to detect human granzyme and performed according to the manufacturer's instructions (BD Biosciences Pharmingen, San Diego, CA).

Reagents.

*TLR agonists:* γ-irradiated Mtb (moi equivalent 500; Mycobacteria Research Laboratories at Colorado State University); Lipoteichoic Acid (10 μg/ml; Sigma); LPS (100 ng/ml; Sigma); CpG DNA (6 μg/ml; Coley Pharmaceuticals); poly I:C (50 μg/ml; Sigma). IFN-γ (Sigma) was used at 10ng/ml. Actinomycin D was used at 10 μM (Sigma). The pan HLA antibody W6/32 (Serotec) and the mouse IgG2a isotype control (Biolegend) were used at 2 μg/ml. Annexin V-APC (BD Biosciences) was used to evaluate apoptosis using flow cytometry according to the manufacturer's instructions.

## Supporting Information

Figure S1 Mtb-Reactive Cells Preferentially Recognize Heavily Infected Cells Dendritic cells (50,000/well) were infected overnight with Mtb H37Rv at a range of multiplicities of infection (range 0–50) and used as APCs to detect IFN-γ responses by CD4-depleted thymocytes (250,000/well) from 4 individual thymocyte donors using the ELISPOT assay.
(191 KB PDF)

Figure S2 Infection with Mtb Induces a Minority of DC to Become Apoptotic Dendritic cells were left untreated, infected with Mtb (moi = 30) or treated with actinomycin D (10 μM), as a positive control for induction of apoptosis, for 18 h. Cells were then stained using Annexin V–APC. The percentage of cells that were Annexin V positive was: 4% for untreated cells (dashed line); 18% for Mtb-infected cells (bold line); and 55% for actinomycin D-treated cells (filled histogram).
(183 KB PDF)

Figure S3 Gamma-Irradiated Mtb Induces Stimulation of DC Dendritic cells (100,000/well) were left untreated or incubated for 18 h with γ-irradiated Mtb H37Rv (moi equivalent 500 to 1), or LPS (100 ng/ml). IL-10 production by DC was detected using an ELISPOT assay.
(182 KB PDF)

## Abbreviations

APC: antigen-presenting cell
CBMC: cord blood mononuclear cells
DC: dendritic cells
ICS: intracellular cytokine staining
moi: multiplicity of infection
Mtb: *Mycobacterium tuberculosis*
PBMC: peripheral blood mononuclear cells
SFU: spot-forming unit
TB: tuberculosis
TLR: Toll-like receptor
TST: tuberculin skin test
