# Supplementary material for: Human Innate Mycobacterium tuberculosis–Reactive αβTCR+ Thymocytes
Source: PLoS Pathog. 2008 Feb 15;4(2):e39. doi: 10.1371/journal.ppat.0040039 (PMC2242840; doi:10.1371/journal.ppat.0040039)
Supplement: Supplementary file 1 [file ppat.0040039.sg001.pdf]

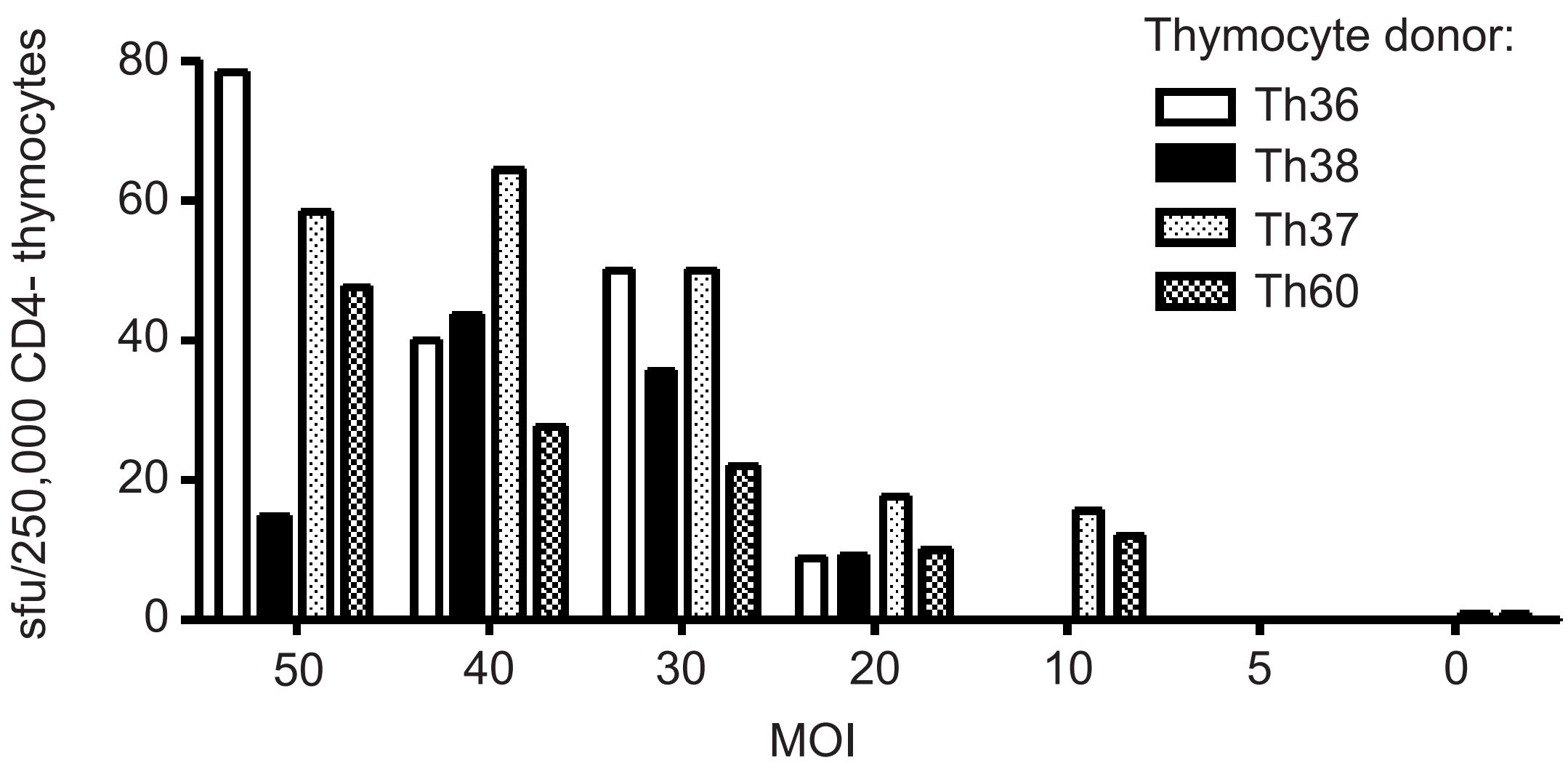

Thymocyte donor:
Th36
Th38
Th37
Th60
sfu/250,000 CD4- thymocytes
80
60
40
20
0
50
40
30
20
10
5
0
MOI
