# Supplementary material for: Human Innate Mycobacterium tuberculosis–Reactive αβTCR+ Thymocytes
Source: PLoS Pathog. 2008 Feb 15;4(2):e39. doi: 10.1371/journal.ppat.0040039 (PMC2242840; doi:10.1371/journal.ppat.0040039)
Supplement: Supplementary file 2 [file ppat.0040039.sg002.pdf]

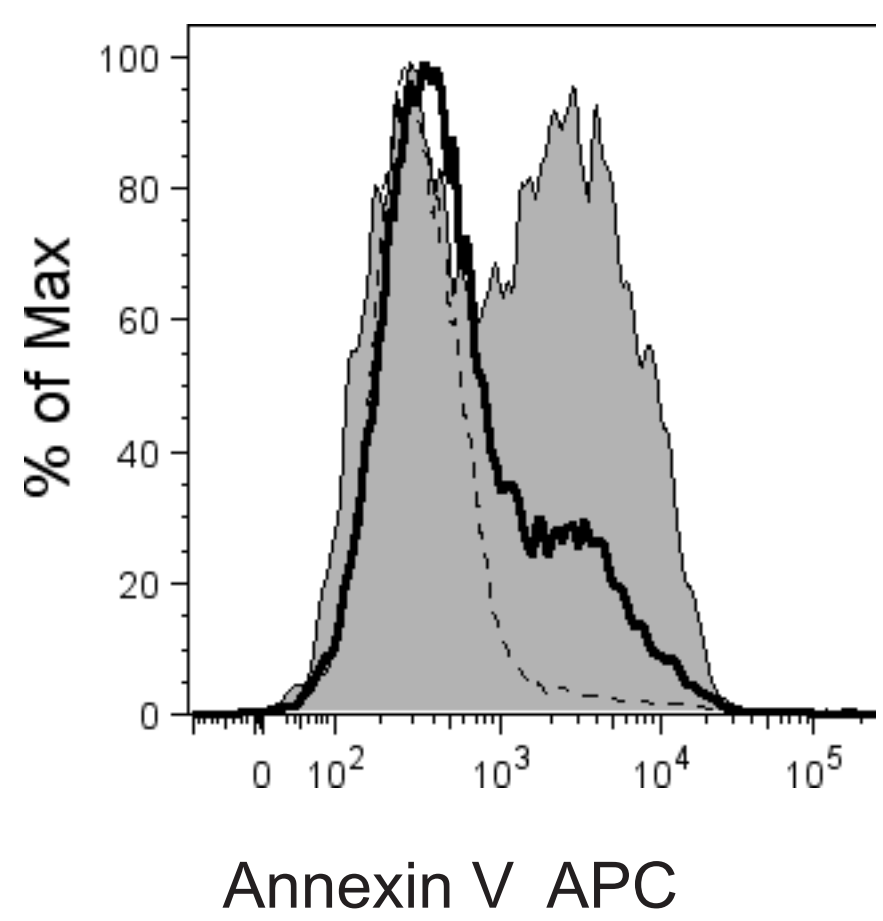

100
80
60
40
20
0
% of Max
0
10^2
10^3
10^4
10^5
Annexin V APC
