# Supplementary material for: Human Innate Mycobacterium tuberculosis–Reactive αβTCR+ Thymocytes
Source: PLoS Pathog. 2008 Feb 15;4(2):e39. doi: 10.1371/journal.ppat.0040039 (PMC2242840; doi:10.1371/journal.ppat.0040039)
Supplement: Supplementary file 3 [file ppat.0040039.sg003.pdf]

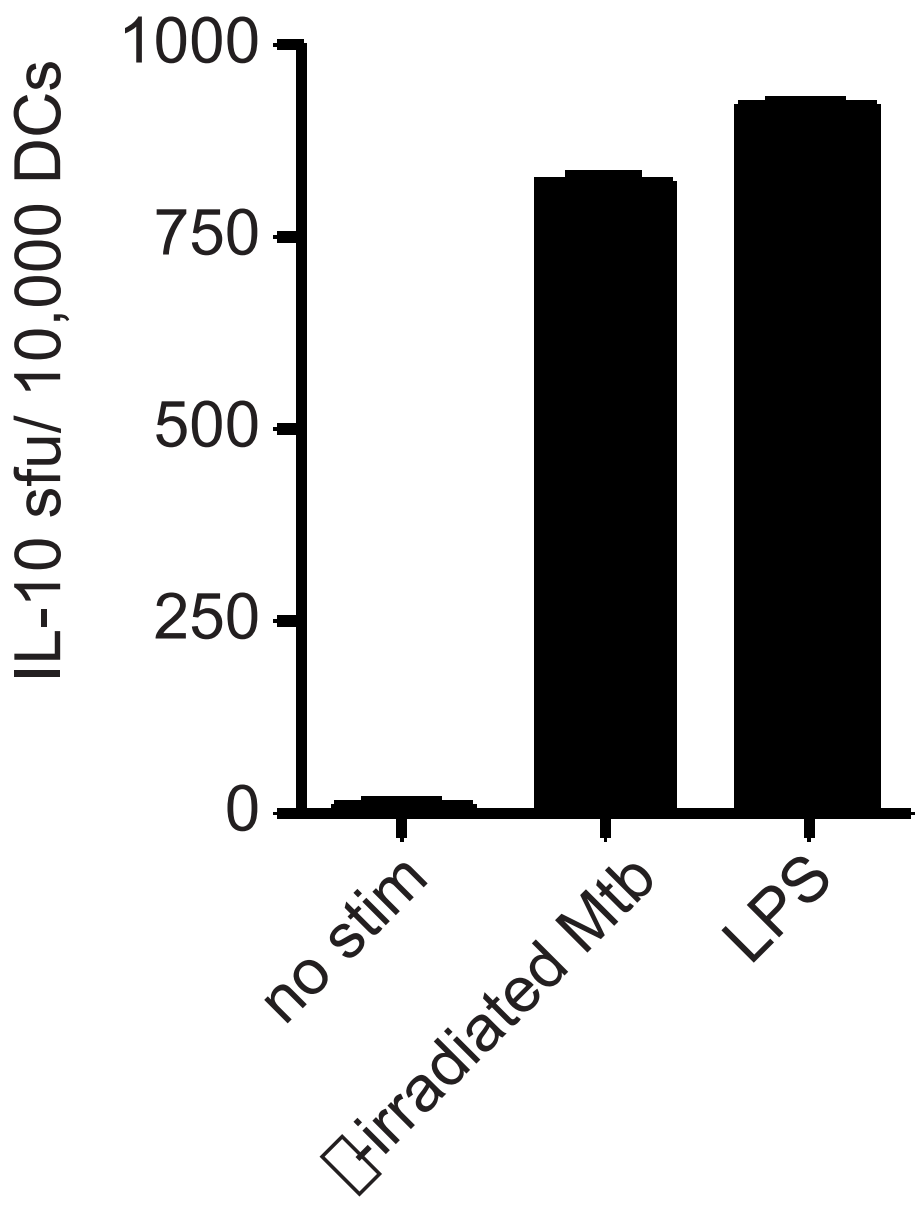

IL-10 sfu/ 10,000 DCs
1000
750
500
250
0
no stim
irradiated Mtb
LPS
